# Production and Characterisation of a Neutralising Chimeric Antibody against Botulinum Neurotoxin A

**DOI:** 10.1371/journal.pone.0013245

**Published:** 2010-10-08

**Authors:** Julie Prigent, Christelle Mazuet, Didier Boquet, Patricia Lamourette, Hervé Volland, Michel R. Popoff, Christophe Créminon, Stéphanie Simon

**Affiliations:** 1 CEA, iBiTec-S, Service de Pharmacologie et d'Immunoanalyse, CEA Saclay, Gif sur Yvette, France; 2 Unité des Toxines et des Bactéries Anaérobies, Institut Pasteur, Paris, France; Universidad Nacional, Costa Rica

## Abstract

Botulinum neurotoxins, produced by *Clostridium botulinum* bacteria, are the causative agent of botulism. This disease only affects a few hundred people each year, thus ranking it among the orphan diseases. However, botulinum toxin type A (BoNT/A) is the most potent toxin known to man. Due to their potency and ease of production, these toxins were classified by the Centers for Disease Control and Prevention (CDC) as Category A biothreat agents. For several biothreat agents, like BoNT/A, passive immunotherapy remains the only possible effective treatment allowing *in vivo* neutralization, despite possible major side effects. Recently, several mouse monoclonal antibodies directed against a recombinant fragment of BoNT/A were produced in our laboratory and most efficiently neutralised the neurotoxin. In the present work, the most powerful one, TA12, was selected for chimerisation. The variable regions of this antibody were thus cloned and fused with the constant counterparts of human IgG1 (kappa light and gamma 1 heavy chains). Chimeric antibody production was evaluated in mammalian myeloma cells (*SP2/0-Ag14*) and insect cells (*Sf9*). After purifying the recombinant antibody by affinity chromatography, the biochemical properties of chimeric and mouse antibody were compared. Both have the same very low affinity constant (close to 10 pM) and the chimeric antibody exhibited a similar capacity to its parent counterpart in neutralising the toxin *in vivo*. Its strong affinity and high neutralising potency make this chimeric antibody interesting for immunotherapy treatment in humans in cases of poisoning, particularly as there is a probable limitation of the immunological side effects observed with classical polyclonal antisera from heterologous species.

## Introduction

Botulinum neurotoxins (BoNTs) are the most toxic substances known [1]. The seven serotypes, BoNT/A to BoNT/G, are produced by different strains of C*lostridium botulinum*. Subtypes have been identified within some serotypes based on amino acid variation from approximately 2 to 32% [2], like BoNT/A1 to BoNT/A4 for serotype A. This anaerobic spore-forming bacterium is ubiquitous in the environment and can germinate under suitable conditions to yield the vegetative bacterium which synthesise the toxin [3]. Human botulism is mainly caused by ingestion of contaminated food with botulinum toxin (foodborne botulism), by contamination of a wound with *C. botulinum* spores (wound botulism) or by intestinal colonization and toxin production in infants <1 year (infant botulism) [4]. *Clostridia* release their neurotoxins as protein aggregates in culture or food. These aggregates, or progenitor toxins, are formed by a complex of an inactive polypeptide toxic chain (150 kDa) and other neurotoxin-associated proteins (haemagglutinin and/or other proteins depending on serotypes) [5], [6] which stabilise neurotoxins [7]. After proteolytic cleavage, the active form consists of a 100 kDa heavy chain (H_C_) linked by a disulfide bridge to a 50 kDa light chain (L_C_). The H_C_ allows the toxin to bind irreversibly to nerve cells at the neuromuscular junction and mediates translocation across the membrane. The L_C_ bears the catalytic activity and, as a Zn^2+^ endopeptidase, cleaves protein member(s) of the SNARE complex involved in the release of acetylcholine [8]. The neuromuscular blockade results in flaccid paralysis [9], generates similar symptoms regardless of BoNT type and may cause death due to respiratory failure or cardiac arrest. Recovery depends on the capacity of new motor axons to reinnervate paralysed muscle fibres. This takes weeks or months according to the quantity and type of toxin [10]. During this period, intensive care is crucial, especially artificial ventilation. Human cases are caused by toxin types A, B and E. Serotype B is the most widely encountered, while serotype A gives the gravest symptoms because of its higher toxicity and longer persistence in the body [11], [12]. The lethal dose of crystalline toxin A is estimated at 1 µg/kg when introduced orally and the dissemination of a single gram could kill more than 1 million people [11].

Because of its extreme toxicity, potency, lethality, ease of production and the lack of an effective treatment, BoNTs have thus been classified by the Centers for Diseases Control and Prevention (CDC) among the 6 major agents (category A) that could be used in bioterrorism [11]. The potential threat of biological warfare and bioterrorism has stimulated renewed efforts to generate vaccines and therapies against agents such as BoNTs. Preventing the effects of such threats requires the development of specific pharmaceutical compounds to protect the general population and the military [13].

Among the different strategies, the use of a protective antibody as a countermeasure appears the most suitable therapy since antibodies are less toxic and more specific than other chemical drugs [14]. Moreover, passive immunotherapy provides immediate protective immunity in the case of emergency after an attack, as compared with vaccination [15]. Two immunotherapies against botulism have reduced botulism mortality rates from approximately 60% to less than 10% [16]. The most frequent antitoxin preparations are equine products such as the bi- or trivalent antitoxin (type AB or ABE) introduced by the FDA in the 1970s [11]. The US Army Medical Research Institute of Infectious Diseases also developed a heptavalent preparation from horse IgG antibodies against serotypes A, B, C, D, E, F and G, with and without their Fc fragment [17]. The other type of antitoxin is the human Botulism Immune Globulin (BabyBIG) approved by the FDA in 2003 as BIG-IV to treat infant botulism caused by type A or B toxins. It was produced from immune plasma of donors who had been immunised with pentavalent (A–E) botulinum toxoid [18]. Although treatments cannot reverse existing paralysis once the toxin has entered the synaptic button, antitoxins can minimise nerve damage, preventing progression of paralysis, and decrease the duration of supportive care [18], [19]. Use of BIG-IV has thus largely reduced hospitalisation costs (by $88 600 per patient). Furthermore, equine antitoxin may cause adverse effects ranging from moderate hypersensitive immune reactions to anaphylactic shock [20]. Protection by therapeutic agents can also differ according to subtype within the BoNT/A serotype. Indeed, reduction in binding affinity and neutralisation between BoNT/A1 and BoNT/A2 has already been noted [21].

Recent publications report the production of mouse monoclonal antibodies (mAbs) with neutralising activity. Most are directed against the H_C_ domain and a recent study described mAbs binding the L_C_ part of BoNT/A [22], [23]. In this context, we have recently produced several mouse mAbs [24], using a recombinant protein corresponding to the C-terminal binding domain of Botulinum neurotoxin A1 (Fc-BoNT/A1, 50 KDa) which has protective antigenic properties [25]. Among the different mAbs neutralising BoNT/A1 *in vivo* [26], the most efficient, murine TA12 (mTA12), was selected to construct a chimeric antibody combining the TA12 variable regions with the constant regions of the human IgG1. The corresponding chimeric TA12 antibody (cTA12) was produced and characterised for further comparison with the initial mouse mAb. Different parameters, i.e. binding affinity, pharmacokinetic parameters and *in vivo* neutralising titre, were determined for both mouse and chimeric antibodies. The results show that the recombinant mAb retained a capacity for highly efficient neutralisation together with a very low dissociation constant (close to 10 pM). Thus, the cTA12 antibody may possibly be considered as a promising candidate of therapeutic tool and could be combined with other mAbs for the treatment of human botulism.

## Results

### Construction of the chimeric monoclonal TA12 antibody

14 monoclonal antibodies were obtained after immunising mice with the recombinant C-terminal domain of BoNT/A1 heavy chain, Fc-BoNT/A1 [24]. 12 out of the 14 antibodies neutralized toxin activity *in vivo* [26]. TA12 mAb, the most efficient antibody, was selected for chimerisation. V_H_ and V_L_ cDNAs were cloned from hybridoma cells secreting TA12 and sequences were verified using scFv production. Functional scFv were selected thanks to their Fc-BoNT/A1 binding, and chimeric light and heavy chain genes were constructed in a specific vector for expression in the baculovirus system or in mammalian cells (see methods).

### Comparison of the chimeric antibody produced in *Sf9* cells and in *SP2/0-Ag14* cells

#### Production in insect cells

**Figure 1:**
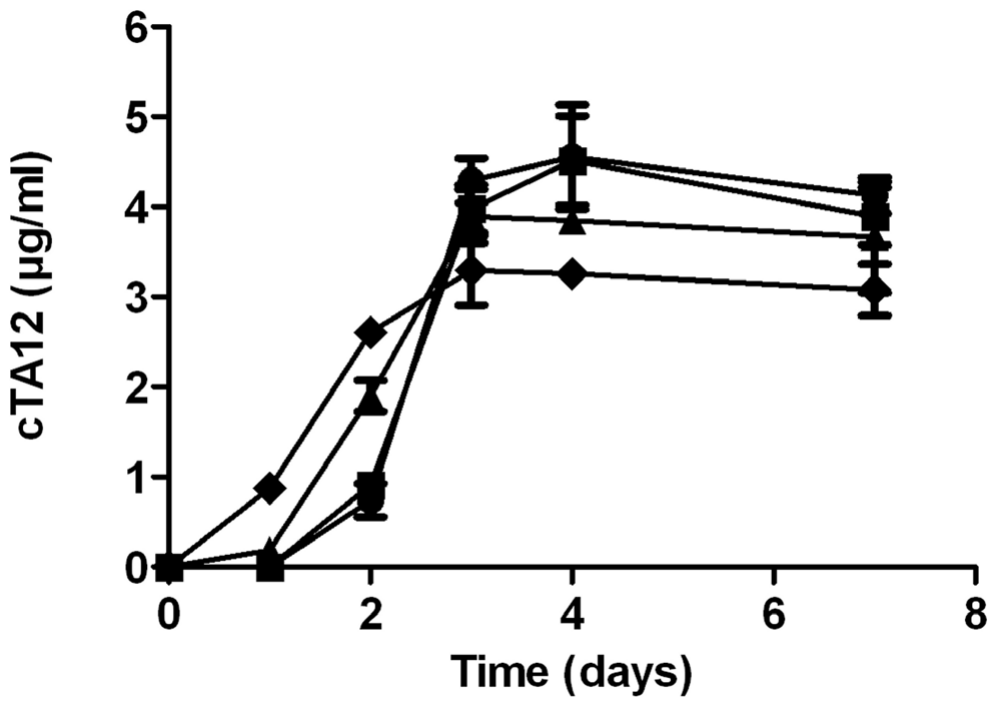
Production of cTA12 in *Sf9* cells by infection of a recombinant baculovirus at different multiplicities of infection (MOI). 1.35×10^7^ cells seeded in T75 flasks were infected at different MOI (•: 0.04; ▪: 0.12; **▴**0.4; ⧫1.2) with the recombinant baculovirus stock. cTA12 containing supernatants were harvested at different times and antibody was quantified by enzyme immunoassay.

**Figure 2:**
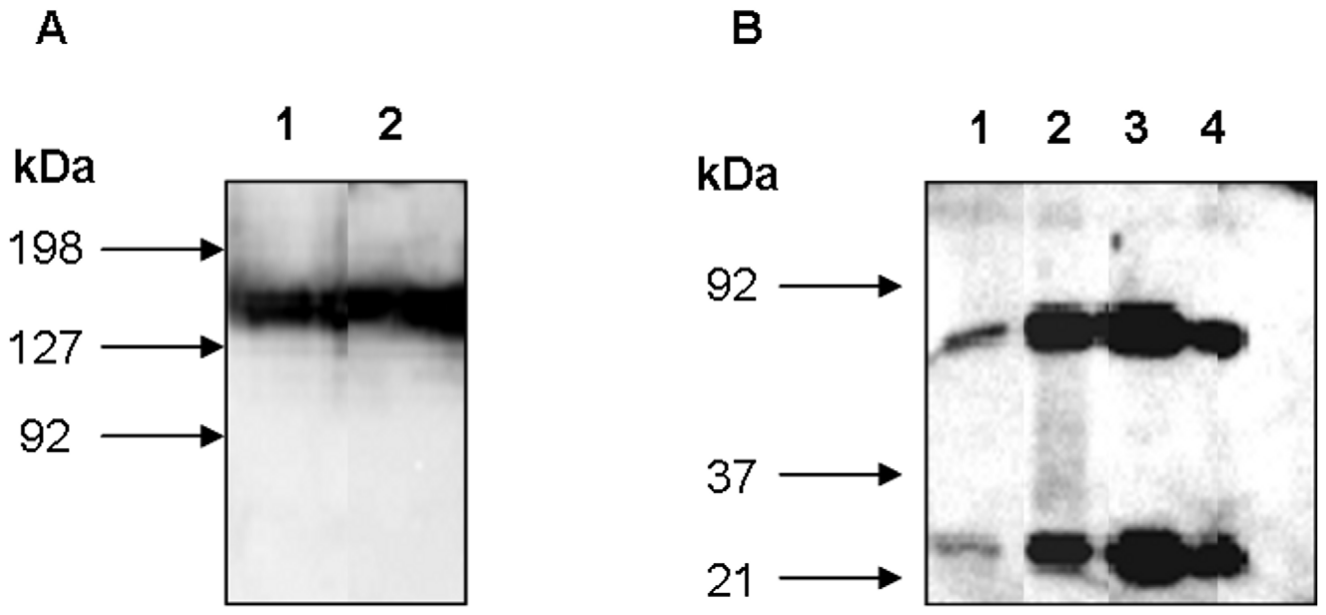
Western blot analysis of cTA12 produced in insect cells and mammalian cells. Samples of TA12 were denatured at 95°C in Laemmli buffer and electrophoresed in 13% SDS-PAGE; in non-reducing conditions (A), lane 1: cTA12 purified from ascitic fluids (clone 16G7) and lane 2: cTA12 purified from Sf9 supernatant; and in reducing conditions (B) Lane 1: Sf9 supernatant, lane 2: cTA12 purified from Sf9 supernatant, lane 3: cTA12 purified from ascitic fluids (clone 16G7), lane 4: unpurified ascitic fluids (clone 16G7). After transfer, membranes were incubated with a polyclonal rabbit anti-human antibody, detected with HRP-conjugated anti-rabbit IgG and revealed using chemiluminescence.

*Sf9* cells were transfected with recombinant bacmids (including genes encoding heavy and light cTA12 chains) to produce recombinant baculovirus. Conditions of antibody production were optimised after a second round of infection and were obtained with cells seeded at a density of 1.8×10^5^ cells/cm^2^ (data not shown). After infection of cells at different MOI (0.04, 0.12, 0.4, 1.2), supernatants were harvested every 24 h and the concentration of secreted cTA12 was measured by EIA using Fc-BoNT/A1 as antigen (Figure 1). The production of cTA12 reached a plateau and thus appeared optimum 3–4 days post-infection. The greater the MOI, the lower the plateau, probably due to the rapid cell lysis. Using purified cTA12 as standard, the concentration of cTA12 in the supernatant was calculated as 5 mg/l (corresponding to 3.75 pg/cell 4 days after infection). The *Sf9* supernatant was analysed by western blotting (Figure 2). The light and heavy chains secreted in the supernatant exhibited approximately the expected 1∶1 stoichiometry (Figure 2B, lane 1). These observations are confirmed by the results obtained after affinity chromatography purification, showing in reducing conditions the heavy and light cTA12 chains (lane 2, Figure 2B) that are correctly assembled into an Ig molecule, since a single band with the expected molecular weight is detected in non-reducing conditions (lane 2, Figure 2A).

#### Production in mammalian myeloma cells

**Figure 3:**
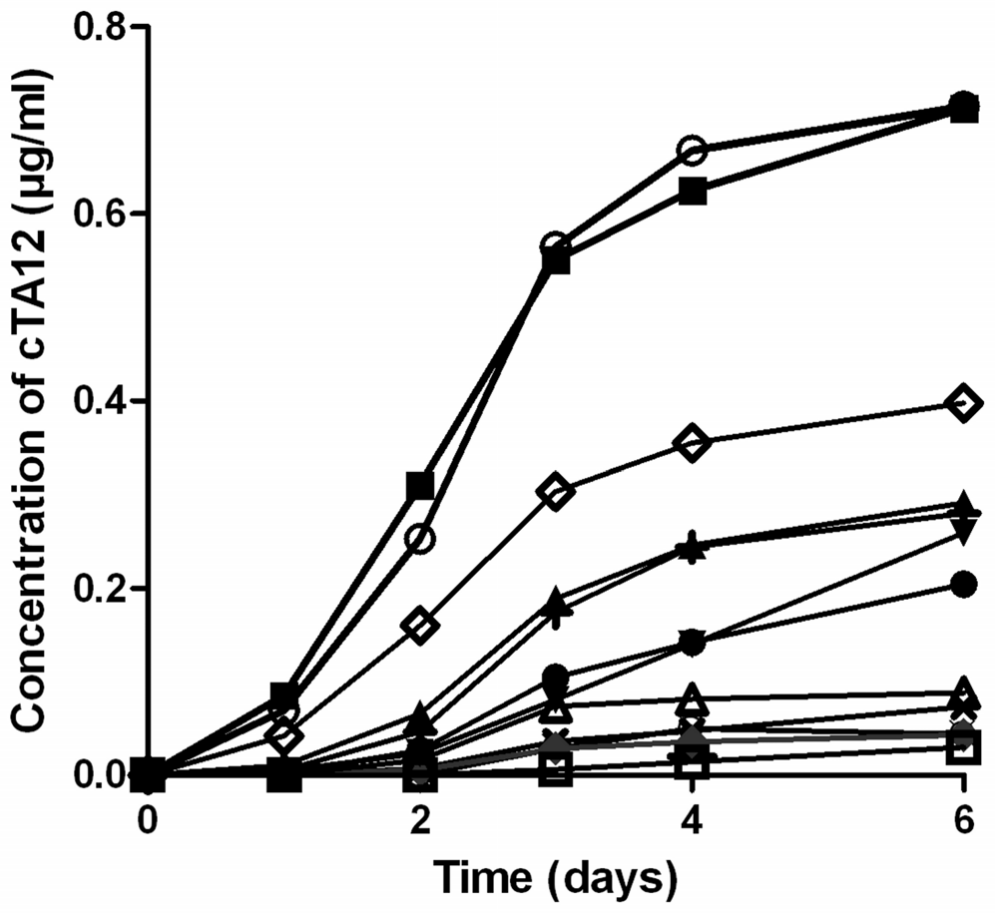
Productivity of different *SP2/0-Ag14* clones secreting cTA12. 6-well plates were seeded with 2.5×10^5^ cells/well in 3 ml of medium culture. The productivity of 12 clones obtained after stable transfection and limiting dilution cloning was tested +12A1; ¤12A2; ▴13E1; **X** 13E4; ⧫13E5; •16C2; □16C4; ▵16G1; ▾16G2; ◊16G3; ○16G6; ▪16G7). Supernatants were harvested every 24 h to measure cell density and cTA12 concentration (by enzyme immunoassay).

Stable expressing *Sp2/O-Ag14* cells were established after co-transfection with the two pcDNA3 plasmids encoding cTA12 light and heavy chains using the limiting dilution cloning method. Screening of the supernatants by EIA allowed the selection of 12 different clones secreting cTA12. A kinetic study of the productivity of these clones was done to evaluate the secreted recombinant cTA12 concentration. As seen in Figure 3, the production curves showed a general pattern similar to that of previous observations with the *Sf9* cells and presenting a maximum at 4 days of culture. The production yields strongly differ according to the clone, probably because of the number of recombinant cDNA copies integrated in the cell genome, the localisation of integration and the ratio between the two cDNA encoding chains. Clones 16G6 and 16G7 proved to be the two best producers, with a production yield (using non-optimised conditions) of 0.8 µg/ml. For 16G7 clones, productivity reached 1 pg/cell at day 4. When optimising the culture conditions, the maximal concentration of functional cTA12 in the 16G7 supernatant reached 1.5 mg/l (data not shown). To increase production of chimeric antibody, 16G7 cells secreting cTA12 were injected into irradiated Balb/c mice for production of ascitic fluids. Total chimeric antibody was evaluated at 1.2 g/l concentration in these ascitic fluids and was purified by affinity chromatography. cTA12 is secreted as a whole antibody (Figure 2A, lane 1,) based on western blot experiments. In the ascitic fluid, the light and heavy chains are thus secreted with a correct 1∶1 stoichiometry and are all assembled into Ig molecules since no fragment other than the two chains was detected using either non-reducing or reducing conditions (Figure 2A, lane 1 and figure 2B, lanes 3 and 4).

### Characterisation of TA12 antibody binding kinetics

**Figure 4:**
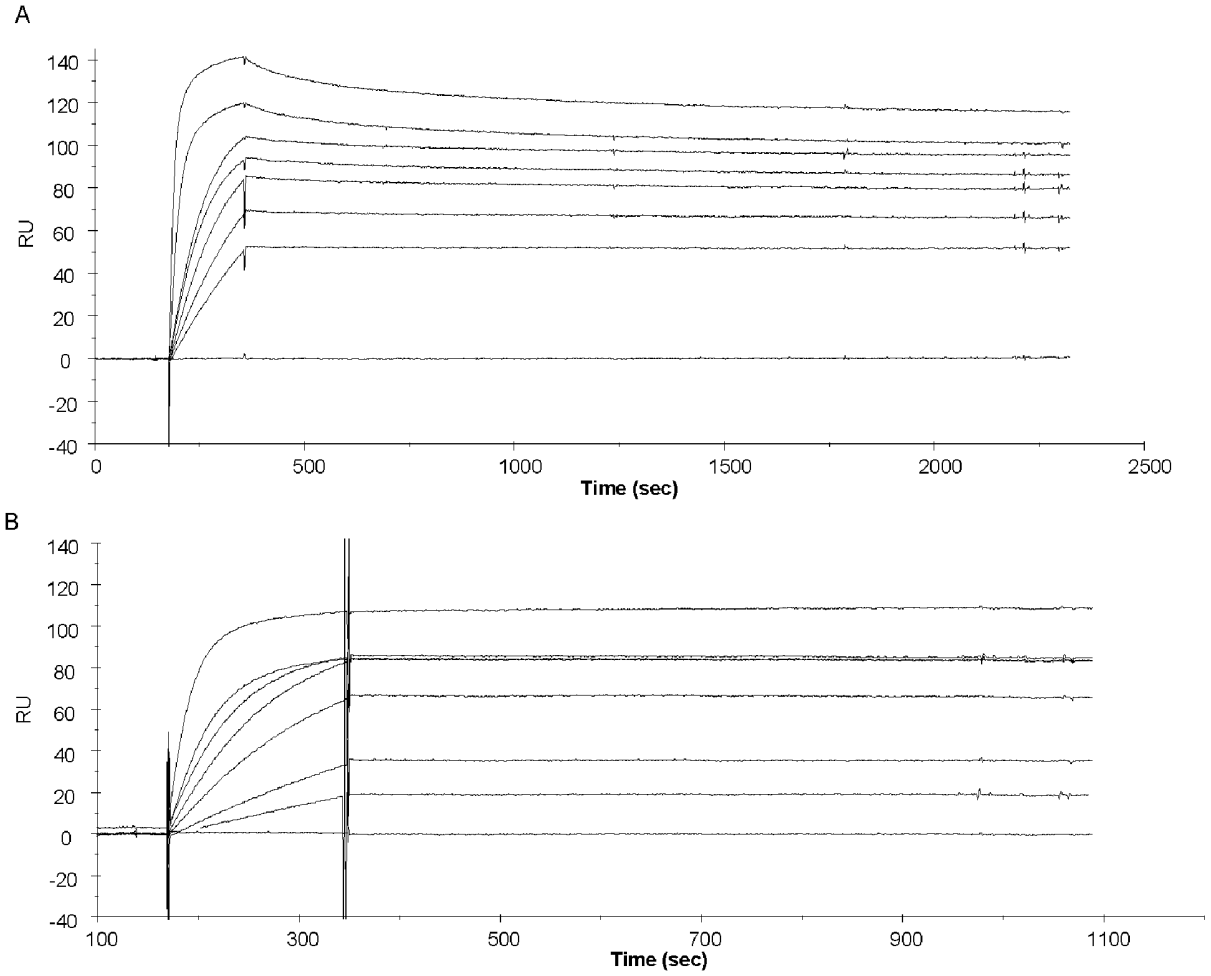
Kinetic analyses of cTA12 and mTA12 binding to Fc-BoNT/A1 by SPR. mTA12 was directly immobilized on a sensor chip at 200 RU (A), and cTA12 was captured at 150 RU using a sensor chip prepared with immobilized anti-human antibody (B). Fc-BoNT/A1 (range 0.8 to 20 nM) was injected at 50 µl/min for 3 min (association phase). Dissociation was monitored over a period of 30 min before the chip was regenerated with 10 mM glycine pH 2. BiaEvaluation Software 3.2 was used to calculate kinetic constants with the Langmuir 1∶1 model fit.

Kinetic parameters of the TA12 mAb were measured by Surface Plasmonic Resonance biosensor technology using Fc-BoNT/A1 as antigen (Table 1). The calculated K_D_ of 15.4 pM for cTA12 produced by *Sf9* cells appears very similar to the value of 14.6 pM obtained for cTA12 produced by murine myeloma cells (ascitic fluids). These sub-nanomolar results are comparable to those obtained for mTA12, which has a K_D_ of 19.1 pM. These observations demonstrate that the chimerisation of TA12 mAb did not modify antibody affinity or kinetics. These strong affinities result partly from a fast association rate constant (k_on_ of 3.7×10^6^ and ≈2×10^6^ M^−1^s^−1^ for mTA12 and cTA12, respectively, see Table 1 and Figure 4) combined with a very low dissociation rate constant (k_off_), particularly for the cTA12 antibody showing a k_off_ value at the limit of detection for the Biacore calculation (Table 1 and Figure 4). This very slow k_off_ allows us to estimate a minimum half-life of 5.12 h at RT for the cTA12/Fc-BoNTA1 complex.

**Table 1:** Association (k_on_) and dissociation (k_off_) rate constants and equilibrium dissociation constant (K_D_) of murine and chimeric TA12 binding to Fc-BoNT/A1.

Antibody	KD (M)×10^−11^	kon (M^−1^.s^−1^)×10^6^	koff (s^−1^)×10^−5^
mTA12	1.74±0.75	3.72±0.21	6.34±2.12
cTA12 (Sf9)	1.54±0.21	1.82±0.81	2.84±1.42
cTA12 (Ascite/*SP2/0-Ag14*)	1.46±0.36	2.26±0.31	2.71±1.30

K_D_ was calculated from k_on_ and k_off_ with n = 4 for all experiments.

### Functionality of the recombinant cTA12

Migration profiles of cTA12 light and heavy chains produced by either insect cells or by myeloma murine cells do not present any differences (Figure 2). However, the chimeric recombinant antibody may be less stable than the murine wild-type mAb and the purification process may also lead to some loss of functional antibody. To evaluate better the possible discrepancy between chimeric antibody concentration measured by UV absorbance and the true corresponding functional concentration, a comparative experiment was designed based on a sandwich enzyme immunoassay taking the murine wild-type antibody as reference (see methods).

**Figure 5:**
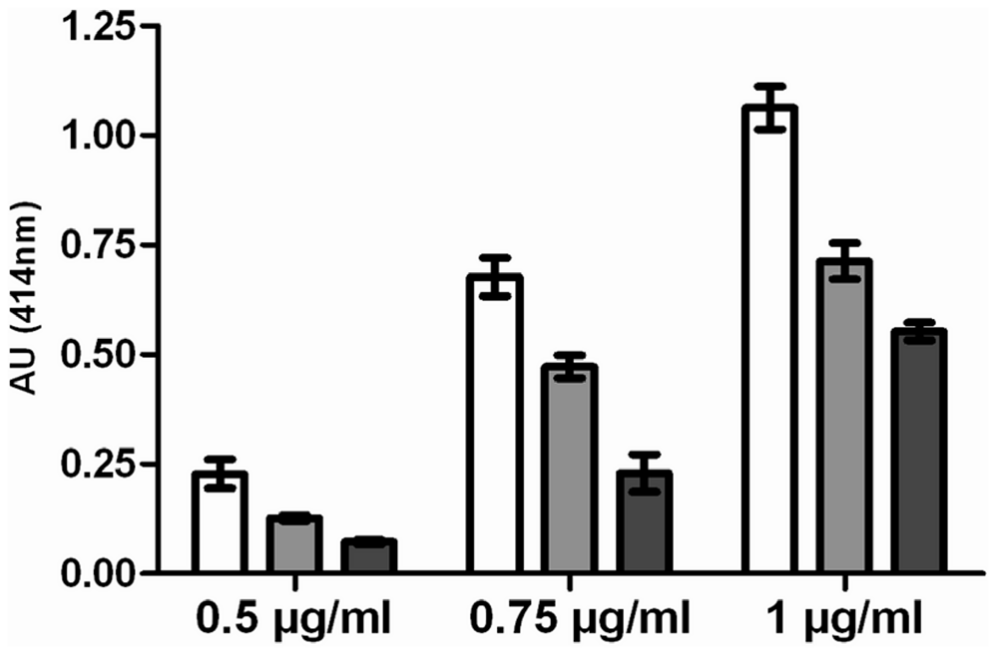
Quantification of functional cTA12 recognising native BoNT/A1 by a sandwich immunoassay. Native BoNT/A1 from culture supernatant (100 µl at 300 LD100) was incubated in 96-well microtitre plates coated with different dilutions of purified TA12 (from 0.05 to 1.5 µg/ml). TA13-labeled AChE was used as the tracer antibody, and absorbance was measured at 414 nm after incubation with AChE substrate. cTA12 purified from ascitic fluids and cTA12 purified from Sf9 supernatant were compared using mTA12 as reference (□ purified mTA12; ▪ cTA12 purified from ascitic fluids; ▪ cTA12 purified from S*f9* supernatant).

Assuming that potential denaturation during adsorption to the solid phase is similar for the two types of purified antibodies, and since the affinity of the two mAbs is identical (as deduced from Biacore experiments), the purified chimeric mAbs (from mammalian and insect cells) were compared with purified mTA12 for their capacity to recognise native BoNT/A1 in quasi equilibrium conditions (considering their kinetic parameters). As shown in Figure 5, for the same UV absorbance, there was a loss of signal of more than 50% for cTA12 purified from insect cell supernatants as compared with mTA12, showing that a major part of the recombinant mAb produced by the insect cells might not be functional. The loss of signal is less important for purified cTA12 from ascitic fluids (close to 30%) Taking into account these results, further experiments were performed with the cTA12 purified from ascitic fluids rather than from *Sf9* culture supernatants.

### Pharmacokinetic studies of murine and chimeric TA12 antibodies in mouse

**Figure 6:**
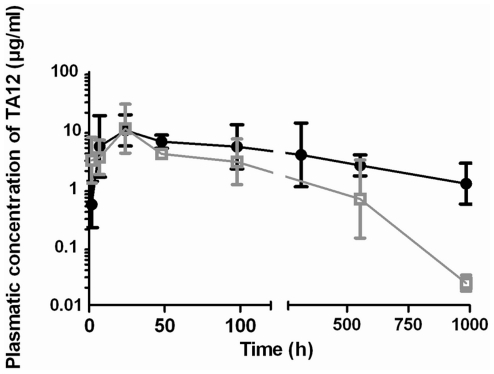
Pharmacokinetic analysis of cTA12 and mTA12 in mice. 50 µg of purified antibody was intraperitoneally injected into Swiss mice (n = 3 or 4). Mice were sacrificed at different times to calculate plasma concentrations of mAb (• mTA12; 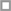 cTA12) using a competitive immunoassay. Data were analysed and fitted with WinNonLin professional software (Pharsight®).

During previous pharmacokinetic studies, mTA12 antibody proved to exhibit a slower clearance than its F(ab′)_2_ counterpart (4 h *vs* 22 days), resulting in a 100-times longer *in vivo* half-life [26]. It thus seemed important to compare the half-life of the murine and chimeric TA12 antibodies. After injecting antibodies intraperitoneally into mice, plasma concentrations of cTA12 and mTA12 were determined at different times by competitive immunoassay. As shown in Figure 6, an initial rapid increase was observed within the first hours after injection for both antibodies, corresponding to antibody transfer from the peritoneum to the blood compartment. A maximum was reached approximately 24 h post-injection, followed by a decrease during the subsequent five weeks. Whereas the plasma concentration appears a bit lower for cTA12 than the mouse mAb during the first day after injection, the maximum concentrations were quite close. However, the plasma clearance of cTA12 was clearly faster than that of the parent mAb, and less than 1% of the maximum concentration remained after 700 h, as compared with about 95% for mTA12. These discrepancies were predictable since the chimeric antibody included a majority of human sequences which further induced faster elimination in the mouse. Nevertheless, values can be included in a pharmacokinetic model (without compartment) to evaluate the half-lives of both antibodies. The cTA12 half-life was close to 7 days, which is 2.5 times shorter than that of mTA12, but much longer than that of the (Fab′_2_) fragments. When used in a more favourable environment such as immunotherapy in humans, the cTA12 half-life should greatly increase and become comparable to that of mTA12 in mouse.

### *In vitro* and *in vivo* neutralisation of the neurotoxin with murine and chimeric antibodies

When studying the ability of mouse and chimeric antibodies to neutralise the neurotoxin, the cTA12 antibody retained the very efficient neutralising power of mTA12 with a neutralisation titre of nearly 13–14 IU/mg, as compared with 6.2 to 20.8 IU/mg for the mouse antibody (Table 2).

**Table 2:** B0NT/A1 neutralisation by the murine and the chimeric TA12 mAb according to the reference method L+10 from the Pharmacopoeia.

Antibody	IU/mg
mTA12	6.2–20.8
cTA12	12.9–14.5

The neutralising activity of TA12 corresponds to a protection of 1,000 estimated MLD_50_ of BoNT/A1 with 50 µg of antibody or to 286 IU/mg according to the method of Hatheway and Dang as described by Mazuet *et al.* [26].

In addition, the protective activity of cTA12 was tested *in vivo* by the co-injection protocol consisting in separate injections of the toxin and antibody without any *in vitro* preincubation step. As shown in Table 3, an intraperitoneal injection of 2.5 µg or 250 ng of cTA12 was able to completely protect all the mice challenged with a lethal injection of 5 estimated MLD_50_/mouse of BoNT/A1. A dose of 25 ng of cTA12 per mouse only partially protected the mice challenged with 5 estimated MLD_50_. The *in vivo* protective activity of cTA12 is identical to the one obtained with mTA12 antibody [26] and comparable to the *in vitro* protective activity against BoNT/A1 (Table 4).

**Table 3:** Neutralization potency of cTA12 using the *in vivo* co-injection protocol.

**cTA12 (ng/mouse)**	2,500	250	25	2.5	0
**surviving mice**	10/10	10/10	5/8	0/10	0/10

The neutralizing activity was determined using the mouse protection assay with 5 estimated MLD_50_/mouse of BoN/A1. BoNT/A1 (0.5 ml) and serial dilutions of cTA12 (0.5 ml) were injected separately into each mouse of a group of 8–10. Results are expressed as surviving mice versus the total number of mice.

**Table 4:** BoNT/A1, BoNT/A2, and BoNT/A3 protection by the murine and chimeric TA12 mAbs.

		mAb quantity (ng/mouse)			
Ab	serotype	2,500	250	50	2.5
**mTA12**	**BoNT/A1**	10/10	10/10	10/10	0/10
	**BoNT/A2**	10/10	3/9	0/9	ND
	**BoNT/A3**	10/10	10/10	0/9	ND
**cTA12**	**BoNT/A1**	10/10	10/10	10/10	0/10
	**BoNT/A2**	10/10	0/10	ND	ND
	**BoNT/A3**	10/10	10/10	5/10	ND

MAb protection activity was determined using the mouse protection assay with 5 estimated MLD_50_/ml of BoNT/A1, BoNT/A2 or BoNT/A3 and serial dilutions of mAbs. 5 estimated MLD_50_/mouse were incubated for 30 min at room temperature with 2,500 to 0.25 ng of Mab and the mixture (0.5 ml) was injected intraperitoneally in each mouse of a group of 8 to 10 mice. Results are expressed as the number of surviving mice versus the total number of mice. ND, not done.

Since variability of the BoNT gene and protein sequence within botulinum neurotoxin subtypes A has been reported and these sequence variations impact on antibody binding and neutralisation [21], the capacity of mouse and chimeric antibodies to neutralise BoNT/A2 and /A3 was evaluated using a mouse neutralisation assay with 5 estimated MLD_50_ per mouse of each toxin subtype. The two forms of antibody showed a marked reduction in neutralising capacity of 10- and 100-fold for BoNT/A3 and /A2, respectively (table 4). These results appear in accordance with published data [21] reporting that mAbs directed against BoNT/A1 showed a 500- to 1,000-fold reduction in binding affinity and a minimal neutralisation capacity for A2 toxin.

## Discussion

There is an urgent need to produce neutralising antibodies devoid of side effects and directed against botulinum neurotoxins, especially the most toxic for humans, the serotype A. In this context, the mAbs produced in our lab against BoNT/A1 for diagnostic purposes were evaluated for their potential neutralising ability [26]. In this recent study, TA12 mAb (mTA12) was characterised as the most powerful due to its great efficiency in neutralising the toxin *in vivo* even alone. Moreover, its high affinity and good half-life highlighted its potential for human immunotherapy purposes.

To limit or avoid possible side effects linked to the murine origin of the Ig [20], a cloning strategy replacing most of the mouse sequences by the human counterparts, i.e. production of a chimeric antibody, was initiated. The chimeric antibody (cTA12) was produced and characterised either in insect cells (*Sf9*) infected with a recombinant baculovirus or in murine myeloma cells (*SP2/0-Ag14*) transfected with recombinant vectors. In the optimal culture conditions, cTA12 reached a concentration of 1.5 µg/ml in the supernatant of *SP2/0-Ag14* cells. This value is of the same order as in a previous publication reporting the production in CHO cells of a chimeric antibody directed against BoNT/A, ranging from 0.5 to 3 µg/ml in the supernatant [27]. For production of chimeric antibody using the baculovirus system, cTA12 reached a concentration of 5 µg/ml, corresponding to the lower part of the range (between 6 and 18 µg/ml) previously published [28]. Without optimising conditions, antibody synthesis per cell was 5-fold greater in *Sf9* cells than in *Sp2/O-Ag14* cells (3.75 pg/cell vs 0.8 pg/cell at day 4). This difference in productivity of the two systems of production is therefore consistent with the literature values.

As *Sp2/O-Ag14* myeloma cells derive from B lymphocytes, cells dedicated to antibody synthesis and secretion, one might expect a greater efficiency of these cells, allowing production of high quantities of antibody. However, due to immortalisation, synthesis might have been attenuated. Moreover, during transfection some bias could have been introduced in the selection of positive clones that was based on two criteria: the production of functional antibody and obviously cell growth. Clones growing very slowly were discarded for practical reasons, even if they may be among the best secreting ones. Even if the antibody yield does not appear in favour of myeloma cells, two other arguments partially counteract the use of insect cells. First, *Sf9* cells do not perform the same post-translational modifications as the mammalian cells, especially for glycosylation. Since modified glycosylated moieties are a well-known source of immune response in mammalian hosts, these may at least shorten the half-life of the antibody in humans, thus probably decreasing the neutralising efficiency, or even worse, inducing immunological side effects, which we try to limit by creating a chimeric antibody. On the other hand, the lytic baculovirus cycle of *Sf9* cells not only releases intracellular proteases that may alter the stability of secreted functional antibodies, but also unfolded or unfinished intracellular chains of antibodies that could interfere with functional antibody purification and qualification. To evaluate the presence of unfinished or degraded fragments of light and heavy chains in cTA12 purified from *Sf9* supernatants or ascitic fluids, western blot experiments were performed in non-reducing or reducing conditions. No degraded antibody was detected, leading to the conclusion that for both production systems these unwanted forms may account for less than 10% of recovered antibody. However, this absence of characterisation of “incomplete” antibody does not guarantee that all synthesised antibody was fully functional, particularly after purification, which could denature recombinant chimeric antibody more than the initial mouse antibody. Functional chimeric antibodies recovered from both production systems were characterised using the same sandwich immunoassay and purified mTA12 as standard (assuming it was 100% functional). Part of the purified cTA12 from baculovirus (up to 70%) appeared not to be functional as compared with mTA12, while most of the chimeric mAb recovered from mammalian system (>70%) appeared functional. The secretion process (particularly post-translational modifications) in insect cells might lower the stability and thus lead to difficulties in maintaining the correct conformation at low pH during purification.

Interestingly, recombinant chimeric antibody retained all the properties of its murine counterpart. First, cTA12 exhibited a remarkably high k_on_ and a k_off_ even lower than that observed for the mouse mAb. The resulting K_D_ was nearly impossible to deduce, since the dissociation rate constant was almost too slow (in the order of 10^−5^s^−1^) to calculate. However, TA12 is clearly a high-affinity antibody with a dissociation constant of at least of 10×10^−12^ M^−1^. This combination of high k_on_ and low k_off_ presents a real advantage in neutralising the target, notably when circulating at low concentrations in the whole body. This high affinity also limits the number of antitoxin injections, thus minimising the potential immune side effects. This advantage is emphasised by the relatively long half-life of the antibody as observed during pharmacokinetic studies. The half-life of the whole mouse antibody is similar to that obtained in a previous study (18.5 days versus 22 days). As expected, the half-life of chimeric TA12 is shorter than that of the mouse mAb in the mouse model (7 days), but much longer than that of the mouse F(ab)′_2_ (4 h). The murine immune system is probably not as favourable as the human one for the chimeric antibody and better stability of cTA12 should be observed after administration to humans. It should be kept in mind that a previous report described a half-life of 6.5 days for the equine antitoxin in a patient [29]. We might expect a longer half life for the present cTA12.

The high affinity of mAb TA12 could explain the strong neutralising effect observed *in vivo*. Correlation between antibody affinity and serum neutralisation has been established for tetanus toxin [30] and verified with anthrax toxin [31]. The better the affinity, the better the neutralisation, whatever the mechanism(s) involved in protection against intoxication using antibodies. Among the 12 neutralising anti-BoNT/A1 mAbs, mTA12 exhibits the strongest neutralisation capacity, evaluated between 6.2 and 20.8 IU/mg, ranking this reagent as a good candidate for immunotherapeutic purposes. The chimeric antibody described here had a neutralising efficiency close to 13 IU/mg. Even if neutralization of BonT/A2 and A3 is less effective, TA12 is still able to neutralise this subtype *in vivo*. This might be improved if this antibody is combined with antibodies neutralising A2 and A3 toxins.

Very promising human or chimeric antibodies have also been described, such as an oligoclonal recombinant antibody, involving 3 mAbs cloned by phage display [32]. This combination inhibits BoNT/A1 with a great potency (45 IU/mg), when each separated antibody was unable to neutralise BoNT/A1. More recently, two other mAbs directed against the H_C_ and the L_C_ parts of BoNT/A proved to protect mice at very low concentrations when they were combined [33]. Two distinct human neutralising antibodies, proved to be efficient alone in the mouse protection assay [22], [34].

However, it seems difficult to compare antibodies with each other, due to the lack of a gold standard for *in vivo* neutralisation tests, in spite of the reference test (L+10) recommended by the European Pharmacopeia [35]. As an illustration, our mTA12 antibody, in the standard mouse neutralisation bioassay, has a neutralising titre of 450 IU/mg of antibody [26], whereas in the L+10 neutralising bioassay the titre falls to 6 to 20 IU/mg.

To the best of our knowledge, TA12 has great potential for therapeutic use, for four main reasons: i) this antibody possesses an extremely low K_D_, which is favourable for immunotherapeutic purposes, ii) its neutralising titre is very high, iii) it can be used alone (and obviously combined with other neutralising antibodies), iv) its long half-life in mice (which should be even better in humans) should avoid repeated injections.

To conclude, the present recombinant chimeric antibody seems to be a good candidate for passive human immunotherapy due to its high affinity in the picomolar range and its *in vivo* neutralising efficiency alone, as characterised by the fast on-rate, very low off-rate and long half-life. This TA12 mAb could be mixed with other chimeric antibodies under development at the CEA (notably anti-BoNT/B and anti-BoNT/E antibodies), to prepare an oligoclonal preparation especially for use against human botulinum intoxication.

## Materials and Methods

### Ethics statement

All experiments were performed in accordance with French and European Community guidelines for laboratory animal handling. The protocols of experiments were approved by the Pasteur Institute (Agreement of laboratory animal use n° 75–279).

### Enzyme Immunoassays (EIAs)

#### Sandwich immunoassays

Two types of sandwich immunoassays were used throughout this study:

Quantitative immunoassay: The concentration of the different forms of TA12 antibody was determined via their capacity to bind the recombinant Fc-BoNT/A1 protein. This EIA was performed in 96-well microtitre plates coated with different antibodies depending on the form of TA12 evaluated. Polyclonal goat anti-mouse IgG or rabbit anti-human IgG antibodies were used (0.6 µg/well) to measure the initial mTA12 or the chimerised one. Supernatants or purified antibodies were incubated overnight at 4°C in EIA buffer (0.1 M phosphate buffer pH 7.4, 0.15 M NaCl, 0.1% BSA and 0.01% sodium azide). After 3 washing cycles (in 10 mM phosphate buffer pH 7.4, 0.1% Tween20), 100 µl/well of the biotinylated Fc-BoNT/A1 (25 ng/ml) was added and the reaction was allowed to proceed for 2 hours (h) at room temperature (RT). After 3 washing cycles, 100 µl/well of streptavidin labelled with 2 EU/ml (for Ellman Unit (EU) definition, see [36]) acetylcholinesterase (AChE) was added for 1-h reaction at RT. After 3 washing cycles, 200 µl of AChE substrate (Ellman's reagent) [36] was added to each well and absorbance was measured at 414 nm after 30-min reaction at RT.

Functional immunoassay: This second sandwich method was used to evaluate the ability of TA12 to bind to the entire native toxin *in vitro*. 96-well microtitre plates were coated overnight at RT with diluted cTA12 or parent mTA12 (in 0.05 M phosphate buffer). After 2 h of saturation in EIA buffer, 100 µl/well of native BoNTA1-containing culture supernatant (at 300 LD100, kindly provided by the Pasteur Institute) was incubated overnight. After 3 washing cycles, 100 µl/well of 5 EU/ml AChE-labelled TA13 (a tracer antibody directed against BoNT/A1 [24]) was added for a 2-h reaction. After 3 washing cycles, 200 µl of Ellman's reagent was added to each well and absorbance at 414 nm was measured after 1.5 h of incubation at RT.

#### Competitive immunoassay

During pharmacokinetic studies, plasma concentrations of mouse and chimeric TA12 antibodies were determined with a competition assay using 96-well microtitre plates coated with TA5, an anti-BoNT/A1 mAb [24]. 100 µl of sample dilutions (plasma dilutions in EIA buffer, ranging from 1∶100 to 1∶1000 for mTA12 and from 1∶5 to 1∶20 for cTA12, or purified antibody for calibration) was added to the wells with 50 µl of 2 EU/ml competitor (mTA12 tracer antibody) and 50 µl of 0.1 nM Fc-BoNT/A1. After an overnight incubation at 4°C, plates were washed before adding 200 µl of Ellman's reagent to each well. Absorbance at 414 nm was measured after 1-h incubation.

### Cell lines

The murine myeloma *SP2/0-Ag14* cell line [37] (a generous gift of Dr. Mazié from the Pasteur Institut [38]) and hybridoma cells secreting mouse TA12 monoclonal antibody were maintained at 37°C with 7% CO_2_, in RPMI medium supplemented with 10% foetal calf serum, 1% glutamine, 1% sodium pyruvate, 1% penicillin/streptomycin. *Sf9* insect cells, derived from *Spodoptera frugiperda* (Invitrogen), were grown as monolayer culture in synthetic InsectExpress medium (PAA). Cells were cultured at 28°C in a humidified incubator. These cells were used for the infection with the recombinant baculovirus.

### Cloning of variable heavy (V_H_) and light (V_L_) regions and construction of recombinant antibody

#### Cloning of V_H_ and V_L_

Total RNA was extracted from mTA12 hydridoma cells (5×10^6^ cells) using the GenElute™ Mammalian total RNA Miniprep kit (Sigma-Aldrich). After preparation of the RNA using the GeneRacer Kit (Invitrogen), reverse-transcription was achieved using heavy chain upstream primer IgG1 (5′-CCAGGAGAGTGGGAGAGGCTCTTCTCAGTATGGTGG-3′) or light chain upstream primer Kappa (5′-CACTACTTCCTGTTGAAGCTCTTGACGATGG-3′). cDNA was then amplified by Race-PCR using the downstream GeneRacer™ primer and the upstream heavy chain primer IgG1 Nested (5′-GGCTCA GGGAAATAGCCCTTGACCAGGCATCC-3′) or light chain primer Kappa Nested (5′-GTGAGTGGCCTCACAGGTATAGC-3′). The V_H_ and V_L_ genes were cloned in pCR2.1 vector with the TOPO TA cloning kit (Invitrogen) and sequenced.

#### ScFv production

After characterising each sequence, specific primers were designed to amplify the entire variable domains V_L_ and V_H_ (V-J and V-D-J regions) for light and heavy chains, respectively. V_L_ and V_H_ products were assembled by overlap extension PCR (SOE-PCR) [39] with a 20-amino-acid linker [Gly_4_Ser]_4_ [40]. Each product was cloned into the SPI 3.0 vector, a prokaryotic expression plasmid derived from the pET26b vector (Novagen, Madison, WI) including extra N-terminal HA-Tag and C-terminal His-Tag for detection and purification purposes [41]. TA12 scFv were further produced using the prokaryotic expression vector SPI 3.0 and the *E. coli* BL21 (DE3) expression strain (Novagen, Madison, WI). Supernatant containing the soluble periplasmic extract was collected and stored at −20°C until use. Periplasm extracts, containing HA-scFv-His products, were screened using two different immunoassays. The first one detects full-length scFv using double Tag detection: an anti-HA-Tag as capture antibody (mAb 12CA5) and an AChE-labelled anti-His-Tag antibody was used as tracer. The second one detects functional scFv using the same coated solid phase, together with biotinylated-FcBoNT/A1 as tracer revealed using AChE-labelled streptavidin. Briefly, 50 µl of periplasmic extract were incubated with 50 µl of tracer antibody in microtitre plates coated with the capture antibody. Comparison of both screening tests allows selection of full-length scFv specific to Fc-BoNT/A, thus presenting correct folding.

### Cloning and production of the chimeric antibody in insect cells using the baculovirus system

DNA encoding variable gene fragments (V_H_ and V_L_) was reamplified by PCR (RedAccuTaq DNA polymerase, Sigma) using oligonucleotides containing restriction sites. PCR products were digested (by XhoI/NheI for V_H_ and SacI/HindIII for V_L_) and inserted in frame with the human heavy chain constant region (IgG1) for V_H_ and the kappa light chain constant region for V_L,_ in pAc-K-CH3 vector (Progen Biotechnik) [28]. Constructions were verified by sequencing. To express the chimeric antibody in the baculovirus system (Bac-to-Bac baculovirus expression system, Invitrogen), chimeric heavy and light chains were subcloned in pFastBacDUAL™ expression vector (Invitrogen), using BamHI and NcoI/PvuII restriction sites for heavy and light chain, respectively. Recombinant bacmids, corresponding to the genetically modified baculovirus (*Autographa californica*) genome containing cTA12 genes, were generated according to the manufacturer's instructions. Briefly, DH10BAC bacterial cells were transformed by the recombinant vector for transposition of the heavy and light chain DNA into the bacmid. Positive recombinant bacmids were used to transfect *Spodoptera frugiperda* (*Sf9*) insect cells for viral particle formation. All procedures for the production of viral particles were performed according to the manufacturer's instructions. Briefly, 0.5 µg of the purified bacmid was complexed with Escort Reagent (Sigma) to transfect 1×10^6^ *Sf9* cells seeded in 6-well plates. 72 h later, the first stock of recombinant baculovirus (P1) containing supernatant was harvested. The titre of the virus was determined by the modified endpoint dilution assay [42]. The virus stock solution was filtered through 0.22 µm filters and stored at 4°C, protected from light. To amplify baculovirus stock, this P1 low titre viral stock was used at a multiplicity of infection (MOI) of 0.1 to infect cells seeded at 2×10^6^ cells/well in 6-well plates or 1.35×10^7^ cells in T-75 flasks. P2 viral stock was harvested 72 h after cell infection.

For antibody production (P3), *Sf9* cells were infected with the high titre (P2) viral stock and 1.35×10^7^ cells in T-75 flasks. Supernatant (containing 1 mM protease inhibitor AEBSF (Fluka)) was harvested from 0 to 7 days post-infection, clarified by centrifugation (5 min at 500g), filtered through 0.22 µm filters and stored at 4°C until purification. To determine the optimal production, 0.2 ml of supernatant was removed every day and the functional antibody concentration was checked by EIA.

### Cloning in mammalian expression vector and production of the chimeric antibody in mammalian cells

Chimeric heavy and light chains from pAc-K-CH3 vector were subcloned separately in pcDNA3 expression vector (Invitrogen) using digestion by BamHI and EcoRV, respectively (further named pcDNA3-H and pcDNA3-L, respectively). *SP2/0-Ag14* cells were co-transfected with pcDNA3-H and pcDNA3-L recombinant vectors using the standard method with Lipofectamine™ reagent (Gibco). To generate stable cell lines secreting the recombinant cTA12 antibody, transfected cells were selected with geneticin (1 mg/ml, Invitrogen) 3 days post-transfection. To assess the production of functional recombinant TA12 antibody, supernatants of geneticin-resistant cells were tested by EIA. EIA-positive cells were cloned by limiting dilution method and supernatants were harvested for the characterisation of antibodies.

### Purification of the recombinant antibody

Recombinant and wild-type antibodies were purified using protein A affinity chromatography [43]. Samples containing the antibody were mixed (1∶1 v/v) with binding buffer (20 mM phosphate buffer pH 7.4, 0.15 M NaCl ) and incubated overnight at 4°C with protein A gel (Millipore). The bound antibody was eluted in 0.1 M sodium citrate buffer (pH 3) and each fraction (750 µl) was neutralised with 250 µl of potassium phosphate buffer (1 M pH 8.8). Fractions containing antibody were pooled and dialysed against 50 mM potassium phosphate buffer pH 7.4, 0.15 M NaCl. The total IgG concentration was determined by measuring UV absorbance at 280 nm (taking as reference 1 mg/ml = 1.4 AU) while the functional IgG was characterised using EIA.

### SDS-PAGE/Western blotting

Cell culture supernatants, ascitic fluids or purified antibodies were denatured in Laemmli buffer (0.25 M Tris-HCl pH 6.8, 4% SDS, 40% glycerol, 0.1% bromophenol blue, and 10% β-mercaptoethanol for reducing conditions) [44] for 5 min at 95°C. After SDS-PAGE (13% gel resolving), proteins were blotted onto PVDF membrane. After saturation in 5% dried milk, membranes were incubated with a rabbit anti-human (RAH) polyclonal antibody (0.5 µg/ml, Jackson Immuno Research) for 1 h at RT. After several washings in PBS-0.1% Tween20, membranes were reacted with a secondary HRP-conjugated anti-rabbit IgG for 20 min and the protein bands were detected via chemiluminescence (ECL, Amersham Biosciences) using a Versadoc apparatus (BioRad).

### SPR analysis

The affinities of mouse and chimeric TA12 antibodies were determined by surface plasmonic resonance (SPR) using a BIAcore 2000 instrument (Biacore, Sweden). All analyses were performed at 25°C on CM5 sensor chip in the running buffer HBS-EP (10 mM Hepes, 0.15 M NaCl, 3 mM EDTA and 0.005% P20 surfactant, pH 7.4). Experiments were performed assuming the existence of a high-affinity antigen/antibody complex [45]. mTA12 antibody was immobilised on the chip at 200 RU using the amine coupling method. cTA12 antibody was indirectly immobilised on a RAH antibody-conjugated chip by injection of a 25 nM solution in running buffer at 5 µl/min for 3 min (to immobilise 150 RU). For both antibodies, kinetic analyses were performed by injecting different concentrations (ranging from 0.8 to 20 nM) of the ligand, Fc-BoNT/A1, for 3 min at 50 µl/min. After 30 min of dissociation, the chips were regenerated with two 30-sec pulses of 10 mM glycine pH 2. The equilibrium dissociation constant (K_D_) was calculated using the ratio between the dissociation rate constant (k_off_) and the association rate constant (k_on_), obtained with global Langmuir 1∶1 fit (BIA evaluation software®, v3.2).

### Pharmacokinetic analyses

Male Swiss mice of 6–8 weeks (body weight from 28 to 32 g) were used for the pharmacokinetic study. 50 µg (100 µl at 500 µg/ml) of 0.22 µm-filtered antibodies was administered intraperitoneally to mice. Blood was collected at 2, 7, 24, 48, 98, 313, 553 and 984 h after injection and centrifuged for 30 min at 3500g at 4°C. Plasma was recovered and stored at −20°C until use. Pharmacokinetic parameters were determined based on the mean antibody concentration values from 3–4 animals per time point. For calculation of *in vivo* blood clearance, data values were fitted using WinNonLin professional software (Pharsight®).

### Mouse protection assay

#### *In vitro* neutralising activity

The ability of the mouse and chimeric antibodies to neutralise the neurotoxin was measured *in vivo* with the mouse lethality assay according to the European Pharmacopeia [35]. For the mouse protection assay, male EOPS mice (Charles River) weighing 16–18 g were used. Botulinum toxin was prepared from culture of *C. botulinum* type A1 strain Hall grown in TGYH medium for four days under anaerobic conditions at 37°C. The culture was centrifuged at 10,000g for 15 min at 4°C to separate bacteria and bacterial debris from the culture supernatant, which was subsequently acidified to pH 3.5, stabilised with 5% sterile glycerol, and stored at 4°C. Neutralising antibodies were assayed by the method recommended in the European Pharmacopeia known as the “L+” test. The test dose determines the relationship between a toxin concentration and a reference antitoxin serum. By definition, one L+ toxin dose is the smallest quantity of toxin, when mixed with one international unit of reference antitoxin, which kills 100% of injected mice by the intraperitoneal route within 96 h. International standard for botulinum type A (NIBSC 59/021) was obtained from NIBSC. The test dose, which is the smallest amount of toxin in a volume of 0.5 ml when incubated with standard serum (representing 0.5 IU/ml with the preparation used in this study) causing the death of a group of four mice (0.5 ml injected intraperitoneally into each mouse), was 5 L+10 (30,000 LD_100_/ml). Variable amounts of mouse and chimeric antibodies diluted in phosphate buffer (pH 6.3) containing 0.2% gelatine (PB-G) were mixed with the test dose (2 ml) and the final volume was adjusted to 5 ml buffer. The mixtures were homogenised and incubated for 1 h at RT. 500 µl of each mixture was injected by intraperitoneal route into each mouse of a group of 4. The mice were observed every day for 96 h. The mixture, containing the largest volume of antitoxin and which fails to protect the mice from death, contained 0.5 IU. Neutralising antibody titres are given as international units per milligram of antibody (IU/mg), one IU of antitoxin being defined as neutralising 10^4^ mouse IP LD_50_ [46].

#### *In vivo* neutralizing activity using the co-injection protocol

The neutralizing capacity of the antibody was evaluated in the *in vivo* mouse lethality test. 5 estimated MLD_50_ of BoNT/A1 in PB-G (0.5 ml) were injected i.p. into Swiss mice weighing 20–22 g (10 mice per group). Concurrently, 2.5 µg of cTA12 (0.5 ml in PB-G) was injected i.p. into a close but different site. Mice were observed and any death was recorded every day during 4 days.

## References

[1] Gill DM (1982). Bacterial toxins: a table of lethal amounts.. Microbiol Rev.

[2] Smith TJ, Hill KK, Foley BT, Detter JC, Munk AC (2007). Analysis of the neurotoxin complex genes in Clostridium botulinum A1–A4 and B1 strains: BoNT/A3, /Ba4 and /B1 clusters are located within plasmids.. PLoS One.

[3] Popoff MR (1995). Ecology of neurotoxigenic strains of clostridia.. Curr Top Microbiol Immunol.

[4] Sobel J (2005). Botulism.. Clin Infect Dis.

[5] Inoue K, Fujinaga Y, Watanabe T, Ohyama T, Takeshi K (1996). Molecular composition of Clostridium botulinum type A progenitor toxins.. Infect Immun.

[6] Hines HB, Lebeda F, Hale M, Brueggemann EE (2005). Characterization of botulinum progenitor toxins by mass spectrometry.. Appl Environ Microbiol.

[7] Chen F, Kuziemko GM, Stevens RC (1998). Biophysical characterization of the stability of the 150-kilodalton botulinum toxin, the nontoxic component, and the 900-kilodalton botulinum toxin complex species.. Infect Immun.

[8] Simpson LL (2004). Identification of the major steps in botulinum toxin action.. Annu Rev Pharmacol Toxicol.

[9] BURGEN AS, DICKENS F, ZATMAN LJ (1949). The action of botulinum toxin on the neuro-muscular junction.. J Physiol.

[10] Foran PG, Mohammed N, Lisk GO, Nagwaney S, Lawrence GW (2003). Evaluation of the therapeutic usefulness of botulinum neurotoxin B, C1, E, and F compared with the long lasting type A. Basis for distinct durations of inhibition of exocytosis in central neurons.. J Biol Chem.

[11] Arnon SS, Schechter R, Inglesby TV, Henderson DA, Bartlett JG (2001). Botulinum toxin as a biological weapon: medical and public health management.. JAMA.

[12] Poulain B, Humeau Y (2003). [Mode of action of botulinum neurotoxin: pathological, cellular and molecular aspect].. Ann Readapt Med Phys.

[13] (2000). Biological and chemical terrorism: strategic plan for preparedness and response. Recommendations of the CDC Strategic Planning Workgroup.. MMWR Recomm Rep.

[14] Casadevall A, Dadachova E, Pirofski LA (2004). Passive antibody therapy for infectious diseases.. Nat Rev Microbiol.

[15] Casadevall A (2002). Passive antibody administration (immediate immunity) as a specific defense against biological weapons.. Emerg Infect Dis.

[16] Shapiro RL, Hatheway C, Swerdlow DL (1998). Botulism in the United States: a clinical and epidemiologic review.. Ann Intern Med.

[17] Dembek ZF, Smith LA, Rusnak JM (2007). Botulism: cause, effects, diagnosis, clinical and laboratory identification, and treatment modalities.. Disaster Med Public Health Prep.

[18] Arnon SS (2007). Creation and development of the public service orphan drug Human Botulism Immune Globulin.. Pediatrics.

[19] Tacket CO, Shandera WX, Mann JM, Hargrett NT, Blake PA (1984). Equine antitoxin use and other factors that predict outcome in type A foodborne botulism.. Am J Med.

[20] Black RE, Gunn RA (1980). Hypersensitivity reactions associated with botulinal antitoxin.. Am J Med.

[21] Smith TJ, Lou J, Geren IN, Forsyth CM, Tsai R (2005). Sequence variation within botulinum neurotoxin serotypes impacts antibody binding and neutralization.. Infect Immun.

[22] Adekar SP, Takahashi T, Jones RM, Al-Saleem FH, Ancharski DM (2008). Neutralization of botulinum neurotoxin by a human monoclonal antibody specific for the catalytic light chain.. PLoS One.

[23] Stanker LH, Merrill P, Scotcher MC, Cheng LW (2008). Development and partial characterization of high-affinity monoclonal antibodies for botulinum toxin type A and their use in analysis of milk by sandwich ELISA.. J Immunol Methods.

[24] Volland H, Larnourette P, Nevers MC, Mazuet C, Ezan E (2008). A sensitive sandwich enzyme immunoassay for free or complexed Clostridium botulinum neurotoxin type A.. Journal of Immunological Methods.

[25] Tavallaie M, Chenal A, Gillet D, Pereira Y, Manich M (2004). Interaction between the two subdomains of the C-terminal part of the botulinum neurotoxin A is essential for the generation of protective antibodies.. FEBS Lett.

[26] Mazuet C, Dano J, Popoff M, Creminon C, Volland H (2010). Characterisation of Botulinum Neurotoxin Type A Neutralizing Monoclonal Antibodies and Influence of Their Half-lives on Therapeutic Activity.

[27] Mowry MC, Meagher M, Smith L, Marks J, Subramanian A (2004). Production and purification of a chimeric monoclonal antibody against botulinum neurotoxin serotype A.. Protein Expr Purif.

[28] Liang M, Dubel S, Li D, Queitsch I, Li W (2001). Baculovirus expression cassette vectors for rapid production of complete human IgG from phage display selected antibody fragments.. J Immunol Methods.

[29] Hatheway CH, Snyder JD, Seals JE, Edell TA, Lewis GE (1984). Antitoxin levels in botulism patients treated with trivalent equine botulism antitoxin to toxin types A, B, and E.. J Infect Dis.

[30] Dokmetjian J, Della VC, Lavigne V, de Lujan CM, Manghi MA (2000). A possible explanation for the discrepancy between ELISA and neutralising antibodies to tetanus toxin.. Vaccine.

[31] Maynard JA, Maassen CB, Leppla SH, Brasky K, Patterson JL (2002). Protection against anthrax toxin by recombinant antibody fragments correlates with antigen affinity.. Nat Biotechnol.

[32] Nowakowski A, Wang C, Powers DB, Amersdorfer P, Smith TJ (2002). Potent neutralization of botulinum neurotoxin by recombinant oligoclonal antibody.. Proc Natl Acad Sci U S A.

[33] Cheng LW, Stanker LH, Henderson TD, Lou J, Marks JD (2009). Antibody protection against botulinum neurotoxin intoxication in mice.. Infect Immun.

[34] Adekar SP, Jones RM, Elias MD, Al-Saleem FH, Root MJ (2008). Hybridoma populations enriched for affinity-matured human IgGs yield high-affinity antibodies specific for botulinum neurotoxins.. J Immunol Methods.

[35] European Pharmacopoeia (2002). Botulinum antitoxin. 4th edition.

[36] Ellman GL, Burkhalter A, Ladou J (1961). A fluorometric method for the determination of hippuric acid.. J Lab Clin Med.

[37] Shulman M, Wilde CD, Kohler G (1978). A better cell line for making hybridomas secreting specific antibodies.. Nature.

[38] Dighiero G, Lymberi P, Mazie JC, Rouyre S, Butler-Browne GS (1983). Murine hybridomas secreting natural monoclonal antibodies reacting with self antigens.. J Immunol.

[39] Horton RM, Hunt HD, Ho SN, Pullen JK, Pease LR (1989). Engineering hybrid genes without the use of restriction enzymes: gene splicing by overlap extension.. Gene.

[40] Krebber A, Bornhauser S, Burmester J, Honegger A, Willuda J (1997). Reliable cloning of functional antibody variable domains from hybridomas and spleen cell repertoires employing a reengineered phage display system.. J Immunol Methods.

[41] Boquet D, Creminon C, Clement G, Frobert Y, Nevers MC (2000). Quantitative measurement of bitagged recombinant proteins using an immunometric assay: application to an anti-substance P recombinant antibody.. Anal Biochem.

[42] Dee KU, Shuler ML (1997). Optimization of an assay for baculovirus titer and design of regimens for the synchronous infection of insect cells.. Biotechnol Prog.

[43] Hober S, Nord K, Linhult M (2007). Protein A chromatography for antibody purification.. J Chromatogr B Analyt Technol Biomed Life Sci.

[44] Cleveland DW, Fischer SG, Kirschner MW, Laemmli UK (1977). Peptide mapping by limited proteolysis in sodium dodecyl sulfate and analysis by gel electrophoresis.. J Biol Chem.

[45] Drake AW, Myszka DG, Klakamp SL (2004). Characterizing high-affinity antigen/antibody complexes by kinetic- and equilibrium-based methods.. Anal Biochem.

[46] Centers for Disease Control and Prevention (1998). Botulism in the United States 1899–1996.

